# Oral health’s role in diabetes risk: a cross-sectional study with sociodemographic and lifestyle insights

**DOI:** 10.3389/fendo.2024.1342783

**Published:** 2024-03-07

**Authors:** Amr Sayed Ghanem, Attila Csaba Nagy

**Affiliations:** Department of Health Informatics, Institute of Health Sciences, Faculty of Health Sciences, University of Debrecen, Debrecen, Hungary

**Keywords:** gingivitis, self-perceived oral health, oral health, diabetes, EHIS, chronic diseases, periodontitis, tooth loss

## Abstract

**Introduction:**

Diabetes, a key chronic non-communicable disease, poses a substantial public health burden. The role of oral health as a determinant in the epidemiology of diabetes mellitus, particularly in the Central Eastern European region, remains underexplored. This research aims to examine the impact of specific oral health parameters, including gum bleeding, active dental caries, tooth mobility, and tooth loss, on diabetes prevalence. Additionally, it seeks to clarify the moderating effects of socio-demographic and lifestyle variables on this relationship.

**Materials and methods:**

Data were extracted from the 2014 and 2019 datasets of the Hungarian European Health Interview Survey, comprising a combined nationally representative sample of 11,429 participants. Descriptive statistics were presented as weighted proportions and unweighted counts, and weighted Pearson’s chi-squared tests were employed for assessing associations and goodness-of-fit. Significant predictors were integrated into weighted multiple logistic regression models for analysis. Sensitivity analysis was then conducted to confirm the robustness of the findings.

**Results:**

The study identified ‘Bad’ self-perceived oral health as a diabetes risk (OR=1.35; 95% CI: [1.04-1.75]), with filled teeth being protective (0.65 [0.51-0.84]). Subgroup analysis revealed higher diabetes odds among individuals with primary education (1.41 [1.02-1.96]) and rural residents with tooth loss from decay (3.54 [1.36-9.19]). The bootstrap analysis with 1,000 iterations reaffirmed the model’s stability and predictive accuracy for diabetes.

**Discussion:**

Enhanced oral health is associated with lower risk factors for diabetes. This research highlights the importance of including oral health measures in comprehensive diabetes management approaches.

## 1 Introduction

### 1.1 Diabetes mellitus: a chronic disease challenge globally and in Hungary

In the international landscape of public health, chronic non-communicable diseases (NCDs) represent a critical area of focus that warrants urgent attention. 74% of all mortalities in 2019 were scripted by NCDs, with ischaemic heart disease taking the spotlight by accounting for 16% of worldwide fatalities. Not to be outdone, stroke and chronic obstructive pulmonary disease also play significant roles, while diabetes has made an ascent into the top 10 causes of death, marking a 70% increase since the dawn of the millennium (1–3).

Diabetes represents a formidable challenge in the realm of public health. Currently, it impacts approximately 10% of the global adult population aged 20-79, accounting for an estimated 537 million individuals. Projections indicate that this number will escalate to 783 million by the year 2045. With health-related expenditures surpassing USD 966 billion and contributing to 6.7 million fatalities, the implications for both medical practice and societal well-being are profound (4–6).

Epidemiological data indicate that the prevalence of diabetes predominantly affects individuals aged 45 to 64 in developing regions, while in developed nations, the majority of diabetes cases are observed in those aged 65 and above (7).

In Hungary, NCDs present an alarming public health situation, with 40% of adults reporting at least one chronic condition, surpassing the EU average of 36%. The nation leads the EU in preventable mortality rates at 326 per 100,000 individuals, primarily due to lung cancer, ischaemic heart disease, and lifestyle risks like smoking. CVDs account for one-third of all deaths, and hypertension prevalence stands at 34% for women and 29% for men. Additionally, diabetes affects 9.1% of adults, contributing to 25.7 deaths per 100,000 population (8–12).

### 1.2 The role of oral health in diabetes: a neglected area of study

Globally, oral health disorders present a penetrating public health concern, impacting an estimated 3.5 billion individuals and eclipsing the collective prevalence of the top five non-communicable diseases. Severe periodontal diseases afflict roughly 19% of the adult population, equating to over 1 billion affected individuals, while dental caries in permanent teeth affect a further 2 billion people (13–15).

In Hungary, approximately one-third of the population is affected by common oral conditions such as gingivitis and periodontitis, signaling a broader public health concern. The prevalence of dental caries stands out within the European context, with Hungary reporting some of the highest rates. These dental issues have significant long-term repercussions for oral health maintenance. Moreover, the prevalence of edentulism, particularly among the elderly, is stark: 30% of those 65 and older, and 40% among the 75 and older cohort, suffer from complete tooth loss. This not only reflects the deteriorating oral health among older Hungarians but also underscores the pervasive socio-economic inequalities influencing health outcomes (16–21).

Hungary exhibits the highest age-standardized incidence rate of oral cavity cancer within the European Union, standing at 11.2 cases per 100,000 individuals. For mortality rates, the figures are notably high as well; with an age-standardized rate of 8.9 per 100,000, Hungary ranks fifth in Europe for male deaths. In contrast, the mortality rate for Hungarian women is 2.4 per 100,000, positioning Hungary as the third highest in Europe. When considering both genders, Hungary’s combined age-standardized mortality rate for oral cavity cancer is the second highest across European nations. These alarming statistics, with approximately 3,000 new diagnoses and more than 1,600 deaths annually, highlight the critical need for comprehensive oral health initiatives in the country (22, 23).

### 1.3 The connection between diabetes and oral health

Diabetes has been shown to worsen the severity and progression of periodontal diseases. On the flip side, periodontal disease can negatively impact glycaemic control and may even contribute to complications in diabetes. Notably, treatment of periodontal disease has been linked to a significant reduction in HbA1c levels within 3–4 months among diabetic patients, highlighting the bidirectional nature of this relationship (24). Systemic reviews also indicate that individuals with both diabetes and periodontitis experience a decline in glycemic control over time and have higher mortality rates, including from cardiovascular causes. Additionally, the presence of periodontitis makes one 3.5 times more likely to develop insulin resistance, attributed to systemic inflammation caused by the bacterial composition of periodontal lesions (25–28).

To succinctly condense the connection between oral health and systemic chronic conditions, with a particular focus on diabetes, it’s crucial to start with foundational oral health indicators. Dental caries, beyond being a plaque retention factor, foster an environment suitable for anaerobic bacterial growth, leading to gingival inflammation, as mentioned by Folayan et al. (2021). This inflammation is pivotal in understanding the oral-systemic health nexus, especially in metabolic disorders like diabetes, a connection further reinforced by Sabharwal et al. (2021) (29).

Periodontal disease progression is marked by distinct clinical features, including gingival bleeding and tooth mobility (30). Gingival bleeding not only acts as an initial symptom of periodontal disease but also serves as a key clinical marker for clinicians, indicative of active periodontal pathology (31). This symptom, denoting disruptions in vascular and connective tissue integrity, is a critical measure for early periodontal disease diagnosis. As the disease advances, tooth mobility emerges, driven by the degradation of key supporting structures such as the periodontal ligament and alveolar bone. This degradation, leading to bone resorption and gingival recession, further compromises tooth stability (32).

Utilizing combined data from the 2014 and 2019 Hungarian European Health Interview Surveys (EHIS), this study focuses on examining the impact of oral health indicators, including gum bleeding, dental caries, tooth mobility, and tooth loss, on the prevalence of diabetes. The objective is to succinctly articulate an understanding of the association between oral health and diabetes, making this study one of the first in the region to use a detailed nationally representative dataset to assess these associations.

## 2 Materials and methods

### 2.1 Study design

This research utilized combined datasets from the 2014 and 2019 iterations of the EHIS. Conducted every five years across EU member states, EHIS provides a comprehensive array of health-related data, encompassing aspects from lifestyle choices to chronic disease prevalence, including a dedicated section on oral health. This extensive dataset offers a rich foundation for assessing the connection between various health variables and oral health metrics (33).

Individualized weighting was implemented to mitigate non-response bias and preserve the representativeness of the sample. This approach adhered to the guidelines set by Eurostat, aiming to align the sample’s demographic characteristics with that of the broader population (34). The dataset, curated by the Hungarian Central Statistical Office and overseen by Eurostat, was acquired through a meticulous stratified sampling method, which validates its relevance to the adult population of Hungary living in household settings.

### 2.2 Sample population and data collection

The combined 2014 and 2019 databases for the EHIS had a sample size of 11,429 participants. The initial goal of the survey was to gather data from a representative group of 12,002 individuals aged 15 and older, living in private homes across 510 different municipalities in Hungary for the 2019 EHIS. For the 2014 survey, the target was 9,431 individuals in 532 municipalities. However, the final dataset comprised 5,603 participants for the 2019 survey and 5,826 for the 2014 survey, resulting in response rates of approximately 47% and 55% respectively. In cases where an adult participant lived with a child aged 6 months to 14 years, the survey also collected additional data specific to the child.

Data acquisition involved both electronic methods and in-person interviews, facilitated by trained field interviewers. To maintain consistency and enable cross-national comparability, a standardized Eurostat questionnaire was used. Participants received personalized invitation letters detailing the study’s objectives, timeline, and various response options. Data collection was carried out between September 16 and December 31, 2019 for the 2019 survey, and between September 15 and December 15, 2014 for the 2014 survey, with interviewers utilizing digital devices for immediate electronic record-keeping (35).

### 2.3 Data treatment and variable specification

Oral health indicators included self-perceived oral health status, categorized into ‘Average,’ ‘Good,’ and ‘Bad.’ Additionally, quantifiable metrics such as the number of teeth extracted due to decay and left unreplaced were delineated into discrete categories, namely ‘None,’ ‘1 to 5,’ ‘6 to 19,’ and ‘More than 20.’ Other dimensions of oral health included the presence of filled teeth, active dental caries, tooth mobility, and gingival bleeding. The composite measure of overall oral health was stratified into ‘Optimal’ and ‘Suboptimal,’ while the time since the last dental visit was segmented into ‘More than a year ago,’ ‘Less than 6 months ago,’ and an intermediate category covering visits that occurred between 6 months to a year ago.

Sociodemographic attributes and lifestyle choices related variables were included into the analysis as well. Age was categorized into three groups: 15-34, 35-64, and 65 or older. Gender was classified as male or female. Area of residence was coded as either rural or urban. Employment status was bifurcated into either employed (active) or unemployed (inactive). Regarding educational attainment, individuals were grouped into three tiers: primary education, secondary education, and tertiary education. Lifestyle factors like alcohol consumption and smoking were dichotomized as either users or non-users. Body Mass Index (BMI) was classified as either normal (≤24.9) or overweight/obese (≥25) (36).

Financial standing was examined through two lenses: a self-assessment as either good, average, or poor, and an objective categorization into quintiles, ranging from first to fifth quintile.

All disease-related variables in the study were categorized into binary outcomes, indicating either the presence or absence of the disease, including the primary outcome of interest, which was diabetes. The type of diabetes was not indicated in the survey; therefore, all types of DM were included in the analysis.

### 2.4 Statistical analysis

Descriptive statistical analyses were carried out using weighted proportions, with p-values employed to assess the significance of associations between variables. These analyses guided the selection of variables for the regression model. Following the finalization of the predictor set, a weighted multiple logistic regression model was constructed. The model’s performance was rigorously validated through a series of diagnostic tests, including goodness-of-fit assessments, to confirm both its fit to the data and predictive accuracy.

The level of statistical significance was set at a p-value below 0.05. This criterion was applied across all analytical procedures to ascertain the meaningfulness of relationships between variables. Results stemming from logistic regression were expressed in terms of odds ratios (ORs), accompanied by 95% confidence intervals (CIs). The entirety of the statistical evaluations was executed using STATA IC Version 17.0 software (37).

### 2.5 Sensitivity analysis

Subset analyses were conducted to evaluate the potential impact of confounding variables such as age, educational attainment, residence area, financial status, and income on the relationships between oral health indicators and the outcome variable. These analyses aimed to identify heterogeneity in the effects of oral health indicators across different demographic groups. Additionally, bootstrapping methods were applied to assess the stability and reliability of the logistic regression estimates. This technique involved resampling the dataset with replacement to generate 1,000 bootstrapped samples, which were then analysed using the original logistic regression model. The bootstrapping approach provided an estimate of the distribution of coefficients and tested the resilience of the study’s findings.

## 3 Results

### 3.1 Sociodemographic and lifestyle characteristics

Age was a key determinant, with the highest diabetes prevalence in the 65+ age group (49.44%, p<0.001). Individuals with only primary education showed a notable prevalence (58.03%, p<0.001), and unemployment was strongly linked to higher diabetes rates (71.41%, p<0.001). Overweight and obese individuals had a significantly higher prevalence of diabetes (81.07%, p<0.001) (**Table 1**).

**Table 1:** Sociodemographic and lifestyle characteristics of study participants in weighted proportions (%) and unweighted numbers (n).

Variable	Category	Diabetes				
		Without diabetes *(n, %)*	With diabetes *(n, %)*	Total *(n, %)*	P value	
**Year**	2014	5349 (50.79)	475 (48.18)	5824 (50.57)	0.126	
	2019	4983 (49.21)	550 (51.82)	5533 (49.43)		
**Gender**	Male	4754 (47.03)	480 (46.97)	5234 (47.02)	0.974	
	Female	5578 (52.97)	545 (53.03)	6123 (52.98)		
**Age groups**	65+	2293 (19.38)	538 (49.44)	2831 (21.92)	**<0.001**	
	15-34	2926 (29.91)	48 (5.43)	2974 (27.84)		
	35-64	5113 (50.71)	439 (45.13)	5552 (50.23)		
**Relationship**	Single	4954 (47.43)	414 (40.76)	5368 (46.87)	**<0.001**	
	In a relation	5266 (52.57)	597 (59.24)	5863 (53.13)		
**Educational attainment**	Primary	4862 (44.97)	599 (58.03)	5461 (46.07)	**<0.001**	
	Secondary	3294 (32.46)	263 (25.99)	3557 (31.91)		
	Tertiary	2175 (22.57)	163 (15.98)	2338 (22.01)		
**Employment**	Unemployed	4965 (45.29)	753 (71.41)	5718 (47.51)	**<0.001**	
	Employed	5361 (54.71)	272 (28.59)	5633 (52.49)		
**Residence**	Rural	3289 (29.44)	305 (27.82)	3594 (29.30)	0.289	
	Urban	7043 (70.56)	720 (72.18)	7763 (70.70)		
**Regions**	Capital	2748 (30.14)	313 (33.03)	3061 (30.38)	0.071	
	Other provinces	7584 (69.86)	712 (66.97)	8296 (69.62)		
**Financial status**	Average	5816 (56.22)	605 (59.10)	6421 (56.46)	**<0.001**	
	Good	2682 (27.63)	201 (20.17)	2883 (27.00)		
	Bad	1683 (16.15)	205 (20.72)	1888 (16.54)		
**Income quintiles**	First	2054 (19.25)	259 (26.09)	2313 (19.82)	**<0.001**	
	Second	2082 (19.47)	247 (22.71)	2329 (19.74)		
	Third	2171 (20.70)	211 (20.08)	2382 (20.65)		
	Fourth	2103 (20.43)	199 (20.13)	2302 (20.41)		
	Fifth	1922 (20.15)	109 (10.99)	2031 (19.37)		
**BMI**	Overweight and Obese	5584 (53.66)	830 (81.07)	6414 (55.97)	**<0.001**	
	Normal	4680 (46.34)	182 (18.93)	4862 (44.03)		
**Smoking**	Smoker	2931 (28.49)	195 (19.35)	3126 (27.72)	**<0.001**	
	Non-smoker	7393 (71.51)	830 (80.65)	8223 (72.28)		
**Alcohol use**	Drinker	7259 (71.59)	615 (60.32)	7874 (70.64)	**<0.001**	
	Non-drinker	3060 (28.41)	410 (39.68)	3470 (29.36)		

Bold values indicate statistical significance (p <0.05) according to weighted Pearson’s chi-squared test.

### 3.2 Health related characteristics

Self-perceived health showed a stark contrast in diabetes prevalence, with those perceiving their health as bad having a 30.47% prevalence (p<0.001). The presence of chronic disease was strongly linked to diabetes, with 94.66% of those with diabetes also having a chronic disease (p<0.001). Other significant findings included a higher prevalence of diabetes among individuals with asthma (8.77%, p<0.001), bronchitis (8.93%, p<0.001), and particularly striking was the prevalence in those with hypertension (74.91%, p<0.001). Additionally, a significant association was found between diabetes and hypercholesterolemia, with 38.68% of diabetic individuals having this condition (p<0.001) (**Table 2**).

**Table 2:** Health-related characteristics of study participants showing weighted proportions (%) and unweighted numbers (n).

Variable	Category	Diabetes			
		Without diabetes *(n, %)*	With diabetes *(n, %)*	Total *(n, %)*	P value
**Self-perceived health**	Average	2843 (26.16)	500 (47.87)	3343 (27.99)	**<0.001**
	Good	6500 (65.30)	214 (21.66)	6714 (61.61)	
	Bad	972 (8.55)	309 (30.47)	1281 (10.40)	
**Has chronic disease**	No	5796 (58.20)	55 (5.34)	5851 (53.69)	**<0.001**
	Yes	4442 (41.80)	969 (94.66)	5411 (46.31)	
**Has asthma**	No	9823 (95.43)	933 (91.23)	10756 (95.08)	**<0.001**
	Yes	494 (4.57)	90 (8.77)	584 (4.92)	
**Has bronchitis**	No	9920 (96.31)	926 (91.07)	10846 (95.87)	
	Yes	395 (3.69)	93 (8.93)	488 (4.13)	**<0.001**
**AMI**	No	10112 (98.20)	943 (92.46)	11055 (97.71)	
	Yes	213 (1.80)	80 (7.54)	293 (2.29)	**<0.001**
**CAD**	No	9914 (96.64)	866 (85.63)	10780 (95.71)	
	Yes	398 (3.36)	153 (14.37)	551 (4.29)	
**Hypertension**	No	7248 (72.29)	249 (25.09)	7497 (68.29)	**<0.001**
	Yes	3059 (27.71)	775 (74.91)	3834 (31.71)	
**Stroke**	No	10107 (98.12)	962 (94.08)	11069 (97.78)	**<0.001**
	Yes	209 (1.88)	62 (5.92)	271 (2.22)	
**Has arthrosis**	No	8399 (83.01)	607 (59.75)	9006 (81.05)	**<0.001**
	Yes	1885 (16.99)	409 (40.25)	2294 (18.95)	
**Has chronic liver disease**	No	10262 (99.55)	1004 (98.74)	11266 (99.48)	**<0.001**
	Yes	50 (0.45)	13 (1.26)	63 (0.52)	
**Has cancer**	No	10120 (98.40)	983 (96.39)	11103 (98.23)	**<0.001**
	Yes	184 (1.60)	36 (3.61)	220 (1.77)	
**Has migraine**	No	9095 (87.81)	882 (86.63)	9977 (87.71)	0.284
	Yes	1229 (12.19)	142 (13.37)	1371 (12.29)	
**Has depression**	No	9860 (95.90)	934 (91.80)	10794 (95.55)	**<0.001**
	Yes	433 (4.10)	85 (8.20)	518 (4.45)	
**Has mental illness**	No	9683 (94.31)	929 (91.96)	10612 (94.12)	**0.003**
	Yes	591 (5.69)	85 (8.04)	676 (5.88)	
**Hypercholesterolaemia**	No	9193 (90.29)	617 (61.32)	9810 (87.85)	**<0.001**
	Yes	1034 (9.71)	394 (38.68)	1428 (12.15)	
**Has arrhythmia**	No	9337 (91.48)	785 (77.34)	10122 (90.29)	**<0.001**
	Yes	956 (8.52)	236 (22.66)	1192 (9.71)	
**Has CVDs**	No	8892 (87.26)	642 (63.47)	9534 (85.25)	**<0.001**
	Yes	1440 (12.74)	383 (36.53)	1823 (14.75)	
**Takes prescription medication**	No	5350 (53.82)	41 (4.56)	5391 (49.65)	**<0.001**
	Yes	4960 (46.18)	983 (95.44)	5943 (50.35)	
**Takes over the counter medication**	No	3858 (35.86)	457 (44.97)	4315 (36.64)	**<0.001**
	Yes	6452 (64.14)	567 (55.03)	7019 (63.36)	
**Had influenza vaccination**	No	7254 (71.31)	538 (52.81)	7792 (69.73)	**<0.001**
	Yes	2936 (28.69)	484 (47.19)	3420 (30.27)	
**Had blood pressure measured**	No	2768 (27.02)	72 (7.11)	2840 (25.33)	**<0.001**
	Yes	7512 (72.98)	953 (92.89)	8465 (74.67)	
**Had cholesterol measured**	No	4647 (46.19)	116 (11.63)	4763 (43.23)	**<0.001**
	Yes	5520 (53.81)	905 (88.37)	6425 (56.77)	
**Had blood glucose measured**	No	4483 (44.38)	63 (6.44)	4546 (41.12)	**<0.001**
	Yes	5710 (55.62)	961 (93.56)	6671 (58.88)	
**Had a stool analysis**	No	5705 (84.75)	548 (78.42)	6253 (84.18)	**<0.001**
	Yes	1039 (15.25)	148 (21.58)	1187 (15.82)	

Bold values indicate statistical significance (p <0.05) according to weighted Pearson’s chi-squared test.

### 3.3 Oral health characteristics

Individuals with poor self-perceived oral health had a higher prevalence of diabetes (35.89% in the ‘Bad’ category, p<0.001) compared to those with better oral health perceptions. The presence of filled teeth correlated significantly with diabetes, where those without filled teeth had a higher prevalence (50.04%, p<0.001). Additionally, diabetes prevalence was notably higher in individuals with mobile teeth (11.56%, p<0.001) and those who had more than 20 permanent teeth missing due to extraction (37.64%, p<0.001). A significant relationship was also observed between diabetes and not having permanent teeth extracted due to decay and replaced (85.06%, p<0.001), and those with prosthetic replacements (64.63%, p<0.001). Furthermore, individuals who hadn’t had a dental checkup in more than a year showed a higher prevalence of diabetes (66.70%, p<0.001) (**Table 3**).

**Table 3:** Oral health characteristics of study participants in weighted proportions (%) and unweighted numbers (n).

Variable	Category	Diabetes			
		Without diabetes *(n, %)*	With diabetes *(n, %)*	Total *(n, %)*	P value
**Presence of active caries**	No	7041 (70.04)	732 (72.74)	7773 (70.27)	0.09
	Yes	3037 (29.96)	266 (27.26)	3303 (29.73)	
**Self-perceived oral health**	Average	3183 (30.11)	369 (35.76)	3552 (30.59)	**<0.001**
	Good	5138 (51.72)	284 (28.35)	5422 (49.74)	
	Bad	1975 (18.17)	370 (35.89)	2345 (19.67)	
**Has gum bleeding when brushing teeth**	No	8558 (83.87)	844 (83.03)	9402 (83.80)	0.507
	Yes	1632 (16.13)	169 (16.97)	1801 (16.20)	
**Has filled teeth**	No	3356 (31.40)	522 (50.04)	3878 (32.98)	**<0.001**
	Yes	6882 (68.60)	491 (49.96)	7373 (67.02)	
**Has mobile teeth**	No	9429 (92.69)	896 (88.44)	10325 (92.33)	**<0.001**
	Yes	780 (7.31)	119 (11.56)	899 (7.67)	
**Number of permanent teeth missing due to extraction**	None	192 (2.73)	10 (1.04)	202 (2.56)	**<0.001**
	1 to 5	4199 (54.08)	252 (27.27)	4451 (51.37)	
	6 to 19	2126 (25.29)	336 (34.05)	2462 (26.18)	
	More than 20	1599 (17.90)	367 (37.64)	1966 (19.89)	
**Had permanent teeth extracted due to decay and not replaced**	No	3134 (32.35)	141 (14.94)	3275 (30.88)	**<0.001**
	Yes	7096 (67.65)	871 (85.06)	7967 (69.12)	
**Presence of prosthetic replacement (bridge, denture, or implant)**	No	5749 (57.56)	348 (35.37)	6097 (55.68)	**<0.001**
	Yes	4529 (42.44)	675 (64.63)	5204 (44.32)	
**Last dental checkup**	More than a year ago	5554 (52.81)	688 (66.70)	6242 (53.99)	**<0.001**
	Less than a year ago	4704 (47.19)	332 (33.30)	5036 (46.01)	
**Has missing permanent teeth**	No	4,295 (42.98)	343 (34.48)	4,638 (42.26)	**<0.001**
	Yes	5,938 (57.02)	671 (65.52)	6,609 (57.74)	

Bold values indicate statistical significance (p <0.05) according to weighted Pearson’s chi-squared test.

### 3.4 Multiple logistic regression models

Females showed lower odds of diabetes than males (OR=0.71; 95% CI: [0.57-0.89]). Age also influenced diabetes risk, particularly in the 15-34 age group compared to those 65+ (1.88 [1.04-3.40]). Financial status was relevant, with individuals in good financial standing having higher odds than those with average status (1.32 [1.01-1.73]). Smoking status indicated higher diabetes odds for non-smokers (1.58 [1.19-2.09]), and normal BMI was associated with lower odds compared to overweight or obese individuals (0.49 [0.38-0.63]). Having a chronic disease significantly increased diabetes odds (4.54 [2.92-7.07]), and poor self-perceived health was associated with higher odds compared to average (1.36 [1.06-1.75]). Hypertension was notably associated with higher odds of diabetes (1.63 [1.27-2.10]). Hypercholesterolemia also showed a strong link, with individuals having this condition more likely to have diabetes (2.11 [1.69-2.64]). Conversely, having a peptic ulcer was associated with lower odds of diabetes (0.46 [0.29-0.75]). Additionally, those taking prescription medication were much more likely to have diabetes (3.52 [2.03-6.10]). A particularly strong association was observed for those who had their blood glucose (BG) measured, indicating significantly higher odds of diabetes (6.51 [3.61-11.74]). Other conditions, such as asthma, bronchitis, AMI, CAD, and mental illness, did not show significant associations with diabetes in this analysis.

Self-perceived oral health was significantly associated with diabetes, where those perceiving their oral health as ‘Bad’ had higher odds compared to those with ‘Average’ perception (1.35 [1.04-1.75]). The number of permanent teeth missing due to extraction did not show a significant association with diabetes across various categories. The presence of filled teeth was associated with lower odds of diabetes (0.65 [0.51-0.84]). Other factors, including having teeth extracted due to decay and not replaced, the presence of prosthetic replacements, the timing of the last dental checkup, gum bleeding when brushing, mobile teeth, the presence of active caries, and having missing permanent teeth, did not show significant associations with diabetes in this analysis (**Table 4**).

**Table 4:** Weighted multiple logistic regression analysis of factors affecting diabetes prevalence.

Characteristics		Diabetes	
		OR (95% CI)	P value
**Year**	2014		
	2019	1.02 [0.97-1.07]	0.38
**Gender**	Male		
	Female	**0.71 [0.57-0.89]**	**0.003**
**Age groups**	65+		
	15-34	**1.88 [1.04-3.40]**	**0.036**
	35-64	1.10 [0.84-1.45]	0.475
**Area of residence**	Rural		
	Urban	1.11 [0.88-1.39]	0.379
**Regions**	Capital		
	Other provinces	0.92 [0.72-1.17]	0.485
**Educational attainment**	Primary		
	Secondary	0.98 [0.76-1.26]	0.853
	Tertiary	0.82 [0.58-1.16]	0.265
**Employment**	Unemployed		
	Employed	0.88 [0.66-1.17]	0.389
**Relationship status**	Single		
	In a relationship	0.90 [0.73-1.11]	0.33
**Financial status**	Average		
	Good	**1.32 [1.01-1.73]**	**0.046**
	Bad	1.09 [0.84-1.42]	0.521
**Income quintiles**	First		
	Second	0.91 [0.68-1.22]	0.528
	Third	0.96 [0.71-1.30]	0.78
	Fourth	1.09 [0.77-1.54]	0.617
	Fifth	0.97 [0.63-1.49]	0.881
**Smoking**	Smoker		
	Non-smoker	**1.58 [1.19-2.09]**	**0.002**
**BMI**	Overweight and Obese		
	Normal	**0.49 [0.38-0.63]**	**<0.001**
**Alcohol use**	Drinker		
	Non-drinker	1.16 [0.93-1.45]	0.179
**Has Chronic disease**	No		
	Yes	**4.54 [2.92-7.07]**	**<0.001**
**Self-perceived health**	Average		
	Good	**0.72 [0.54-0.96]**	**0.024**
	Bad	**1.36 [1.06-1.75]**	**0.017**
**Has asthma**	No		
	Yes	0.85 [0.60-1.20]	0.352
**Has bronchitis**	No		
	Yes	0.93 [0.66-1.31]	0.675
**AMI**	No		
	Yes	1.13 [0.74-1.72]	0.576
**CAD**	No		
	Yes	1.19 [0.87-1.64]	0.273
**Hypertension**	No		
	Yes	**1.63 [1.27-2.10]**	**<0.001**
**Had stroke**	No		
	Yes	0.90 [0.60-1.37]	0.625
**Has arthrosis**	No		
	Yes	0.86 [0.69-1.07]	0.184
**Has cancer**	No		
	Yes	0.74 [0.44-1.26]	0.268
**Has migraine**	No		
	Yes	1.06 [0.78-1.42]	0.721
**Has depression**	No		
	Yes	0.95 [0.65-1.40]	0.8
**Has mental illness**	No		
	Yes	0.86 [0.58-1.28]	0.462
**Hypercholesterolaemia**	No		
	Yes	**2.11 [1.69-2.64]**	**<0.001**
**Has arrhythmia**	No		
	Yes	0.92 [0.71-1.19]	0.523
**Has peptic ulcer**	No		
	Yes	**0.46 [0.29-0.75]**	**0.002**
**Takes prescription medication**	No		
	Yes	**3.52 [2.03-6.10]**	**<0.001**
**Takes over the counter medication**	No		
	Yes	0.89 [0.72-1.09]	0.266
**Had influenza vaccination**	No		
	Yes	1.15 [0.94-1.41]	0.173
**Had blood pressure measured**	No		
	Yes	0.70 [0.45-1.08]	0.109
**Had cholesterol measured**	No		
	Yes	0.70 [0.44-1.11]	0.127
**Had blood glucose measured**	No		
	Yes	**6.51 [3.61-11.74]**	**<0.001**
**Had a stool analysis**	No		
	Yes	0.94 [0.73-1.20]	0.606
**Self-perceived oral health**	Average		
	Good	0.90 [0.70-1.16]	0.424
	Bad	**1.35 [1.04-1.75]**	**0.024**
**Number of permanent teeth missing due to extraction**	None		
	1 to 5	1.71 [0.56-5.18]	0.345
	6 to 19	1.58 [0.51-4.87]	0.424
	More than 20	1.46 [0.47-4.57]	0.515
**Had permanent teeth extracted due to decay and not replaced**	No		
	Yes	1.01 [0.72-1.43]	0.954
**Presence of prosthetic replacement (bridge, denture, or implant)**	No		
	Yes	0.99 [0.77-1.26]	0.912
**Last dental checkup**	More than a year ago		
	Less than a year ago	0.89 [0.71-1.13]	0.34
**Has gum bleeding when brushing teeth**	No		
	Yes	0.80 [0.60-1.06]	0.127
**Has filled teeth**	No		
	Yes	**0.65 [0.51-0.84]**	**0.001**
**Has mobile teeth**	No		
	Yes	0.97 [0.70-1.34]	0.859
**Presence of active caries**	No		
	Yes	1.07 [0.83-1.37]	0.621
**Has chronic liver disease**	No		
	Yes	0.82 [0.34-1.98]	0.666
**Has missing permanent teeth**	No		
	Yes	0.90 [0.71-1.14]	0.398

Bold values indicate statistical significance (p <0.05). The ORs are adjusted for other variables in the model.

### 3.5 Sensitivity analysis

The following subpopulations exhibited distinct associations with diabetes. Poor self-perceived oral health in those with primary education (1.41 [1.02-1.96]), rural residents with teeth extracted due to decay (3.54 [1.36-9.19]), and urban residents with poor oral health perceptions (1.43 [1.04-1.96]) showed increased odds. Financially disadvantaged individuals with poor oral health (2.12 [1.19-3.77]), along with overweight and obese populations reporting poor oral health (1.43 [1.07-1.92]), were similarly at higher risk. These results highlight poor self-perceived oral health as a consistent risk indicator for diabetes.

The bootstrap analysis, with 1,000 iterations, validated the initial model’s robustness. Significant variables consistently retained their significance, affirming the stability of the associations and the model’s predictive reliability for diabetes within the study population.

## 4 Discussion

This study’s findings indicated an association between self-perceived poor oral health and a higher likelihood of diabetes. Individuals with filled teeth were less likely to have diabetes, suggesting a protective effect. However, other oral health factors such as tooth loss due to extraction, presence of prosthetics, timing of dental checkups, and symptoms like gum bleeding, tooth mobility, and active caries, did not show a significant connection with diabetes. The study also revealed that certain subpopulations, including those with primary education, rural and urban residents with poor oral health, financially disadvantaged individuals, and overweight or obese people perceiving their oral health negatively, were at a higher risk of diabetes.

### 4.1 Oral health related factors of diabetes

A constant discussion in the scientific literature is how good self-perceived oral health is as a proxy measure for objective oral health. Numerous studies have been conducted in that regard, studies by Atala-Acevedo et al. (2023) and Nascimento et al. (2020) provide pivotal insights. Atala-Acevedo et al. highlighted the correlation between negative self-perceived oral health (SPOH) and clinical indicators of poor oral health, suggesting its utility in dental health program planning (38). Nascimento et al. extended this scope, showing that very poor SPOH is significantly linked with broader socio-economic and general health issues, alongside specific oral health conditions (39). These studies align with the current study’s results, where SPOH was identified as a critical factor in both univariate and logistic regression models, specifically in the context of diabetes risk. This congruence underscores the significance of SPOH not just as an indicator of objective oral health status, but also as a reflective measure of overall health, including its impact on diabetes, thereby validating its inclusion as a comprehensive determinant in diabetes-related epidemiological research.

It is crucial to consider underlying sociodemographic and lifestyle related factors when looking at self-perceived oral health. Subpopulation analysis revealed that individuals with limited education and bad SPOH have an increased diabetes risk. Lower education is usually associated with limited health literacy that could potentially impact oral health, therefore increasing the risk for diabetes (40). Urban residents with a bad SPOH also had an increased risk that could possibly be tied to the fast-paced lifestyle, stress, limited opportunities for physical activity and an increased prevalence of fast food and western diet, therefore negatively impacting diabetes risk (41). Financial constraints play a pivotal role in health outcomes. Economically disadvantaged groups often face barriers in accessing quality healthcare, including dental services, and may have diets that are less conducive to good oral health, indirectly elevating their risk for diabetes (42, 43). Similarly, the correlation between poor SPOH and higher diabetes odds in overweight and obese populations could be rooted in systemic factors like inflammation and poor nutritional choices that are common to both conditions (44, 45).

The increased risk of diabetes associated with tooth loss, as highlighted in the subpopulation analysis for rural residents with teeth extracted due to decay, aligns with existing literature that acknowledges periodontal involvement as a risk factor for diabetes. This association is further corroborated by the findings of Sandra Aremy López-Gómez et al. (2020), who reported a higher mean number of missing teeth in diabetes patients, along with a correlation between tooth loss and socio-economic variables (46). Lotte P. M. Weijdijk et al. (2022) and J M Liljestrand et al. (2015) also support this connection, with Weijdijk et al. identifying a moderately higher risk of tooth loss in diabetes patients (47). Meanwhile, Liljestrand et al. suggest that missing teeth could be a valuable indicator for assessing the risk of not only cardiovascular diseases but also diabetes and overall mortality (48). This finding underscores the reciprocal relationship between oral health indicators and diabetes, illustrating how each can significantly influence the other. It highlights the bidirectional nature of this association, where poor oral health can exacerbate diabetes risk, and conversely, diabetes can contribute to deteriorating oral health.

The inverse relationship between filled teeth and diabetes risk can be explained by several factors, with a primary focus on prevention of tooth loss. Fillings, as a form of preventive dental care, halt the progression of dental caries, thereby reducing the likelihood of tooth loss, a known risk factor for diabetes. This connection is supported by research, such as the study by Ira Lamster et al. (2022), which emphasizes the link between preventive dental care and improved health outcomes in individuals with diabetes (49). The rationale behind this association extends beyond mere dental intervention. Individuals who receive fillings may be more proactive in maintaining oral hygiene and seeking regular dental care, behaviors that correlate with overall health consciousness. This health-aware lifestyle often includes better dietary choices, regular physical activity, and adherence to medical advice, all of which are crucial in managing diabetes risk. Furthermore, the treatment of dental caries with fillings may help mitigate systemic inflammation (29), a condition commonly associated with both poor oral health and diabetes. Untreated dental caries can lead to chronic oral infections, contributing to systemic inflammatory responses. By addressing caries early through fillings, the inflammatory burden on the body may be reduced, subsequently lowering diabetes risk.

### 4.2 Influence of other health related factors on diabetes

Hypertension’s link to diabetes is rooted in shared pathophysiological pathways such as insulin resistance and endothelial dysfunction, with studies indicating that hypertension is twice as common in diabetic patients in comparison to non-diabetics (50). This commonality explains why hypertension was a significant risk factor for diabetes in the regression model, with insulin resistance contributing to increased blood pressure (51). Similarly, hypercholesterolemia’s association with diabetes is characterized by elevated LDL cholesterol levels, which impair insulin sensitivity and beta-cell function, pivotal in diabetes development, explaining its contribution to diabetes risk in the current study (52). Additionally, the presence of peptic ulcers, a significant predictor in this study, intersects with diabetes through shared risk factors like medication use, stress, and susceptibility to infections. Diabetes can exacerbate peptic ulcers due to its impact on gastric motility and heightened infection risk, especially *H. pylori* infection (53, 54).

The significant association of taking prescription medication and regular blood glucose monitoring with increased diabetes risk can be interpreted through clinical and epidemiological perspectives. The correlation with prescription medication likely reflects indication bias, where the need for medication is an indicator of underlying health conditions, such as hypertension or dyslipidaemia, which are risk factors for diabetes. This implies that medication use is more a marker of a predisposed health profile rather than a direct causative factor. Similarly, regular blood glucose monitoring is often more common among individuals at high risk or with prediabetes, indicating pre-existing metabolic imbalances or familial predispositions.

### 4.3 Biological plausibility of findings

The biological plausibility of the observed associations between poor self-perceived oral health and increased diabetes risk can be primarily attributed to the systemic inflammatory response triggered by chronic periodontal disease. Inflammatory mediators released in gum diseases, such as cytokines, can exacerbate systemic insulin resistance and impaired glucose metabolism, mechanisms central to diabetes development (29). This pathway explains the higher diabetes odds linked with ‘Bad’ self-perceived oral health. Additionally, filled teeth, indicating restorative dental care, may reduce oral inflammation and bacterial load, thereby potentially lowering diabetes risk. In subpopulations, limited health literacy, advanced periodontal disease due to lack of dental care, and prolonged inflammatory conditions in financially disadvantaged groups could further accentuate this risk (43), underscoring the connection between systemic inflammation from poor oral health and the pathogenesis of diabetes.

### 4.4 Strengths and limitations

Using data from the Hungarian EHIS, the study benefits from a large, representative sample size. The methodological approach, which includes robust logistic regression models and sensitivity analyses, is designed to account for various socioeconomic and demographic factors, enhancing the external validity of the findings. This careful consideration of multiple confounders and the comprehensive dataset underpin the study’s significance in its regional context.

This study has limitations inherent to its cross-sectional design, particularly in its capacity to establish causality and temporal relationships. The reliance on self-reported data raises concerns of recall bias and subjectivity, affecting the precision of self-perceived metrics like comorbidities and BMI. This might lead to inaccuracies in reporting oral health conditions and diabetes status, potentially skewing the study’s findings on their correlation. Additionally, the lack of distinction between varying severities of chronic diseases and the absence of key biochemical parameters such as glycemia and HbA1c limit the depth of analysis regarding metabolic and renal implications in diabetes outcomes. Diabetes is a heterogeneous condition, variations in disease severity may result in different associations with oral health.

## 5 Conclusion

This research intended to address a notable gap in the literature by investigating the connection between diabetes and oral health within the context of Hungary and Central Eastern Europe. It underscores the critical role of oral health management not only in reducing diabetes risk but also in mitigating complications and improving disease indicators in diagnosed patients. The findings advocate for the regular evaluation of diabetic patients by dental professionals to address oral health issues effectively. Moreover, the study highlights the importance of managing comorbid conditions alongside oral health, emphasizing an integrated approach to patient care in this region. Future studies employing longitudinal designs are suggested to explore the causal relationships and temporal dynamics between oral health and diabetes.

## Data availability statement

The data analyzed in this study is subject to the following licenses/restrictions: The data presented in this study are available upon request from Hungarian Central Statistical Office who performed and supervised the data collection. Requests to access these datasets should be directed to www.ksh.hu/?lang=en.

## Author contributions

AG: Conceptualization, Formal Analysis, Methodology, Visualization, Writing – original draft. AN: Conceptualization, Supervision, Writing – review & editing.
